# The Role of CXC Chemokines in Cardiovascular Diseases

**DOI:** 10.3389/fphar.2021.765768

**Published:** 2022-05-20

**Authors:** Xiyi Lu, Zhen Wang, Di Ye, Yongqi Feng, Menglin Liu, Yao Xu, Menglong Wang, Jishou Zhang, Jianfang Liu, Mengmeng Zhao, Shuwan Xu, Jing Ye, Jun Wan

**Affiliations:** Hubei Key Laboratory of Cardiology, Department of Cardiology, Renmin Hospital of Wuhan University, Cardiovascular Research Institute, Wuhan University, Wuhan, China

**Keywords:** CXC chemokines, cardiovascular disease, atherosclerosis, hypertension, myocardial infarction and rejection after heart transplantation

## Abstract

Cardiovascular disease (CVD) is a class of diseases with high disability and mortality rates. In the elderly population, the incidence of cardiovascular disease is increasing annually. Between 1990 and 2016, the age-standardised prevalence of CVD in China significantly increased by 14.7%, and the number of cardiovascular disease deaths increased from 2.51 million to 3.97 million. Much research has indicated that cardiovascular disease is closely related to inflammation, immunity, injury and repair. Chemokines, which induce directed chemotaxis of reactive cells, are divided into four subfamilies: CXC, CC, CX3C, and XC. As cytokines, CXC chemokines are similarly involved in inflammation, immunity, injury, and repair and play a role in many cardiovascular diseases, such as atherosclerosis, myocardial infarction, cardiac ischaemia-reperfusion injury, hypertension, aortic aneurysm, cardiac fibrosis, postcardiac rejection, and atrial fibrillation. Here, we explored the relationship between the chemokine CXC subset and cardiovascular disease and its mechanism of action with the goal of further understanding the onset of cardiovascular disease.

## Introduction

Cardiovascular disease is a long-standing major health problem. Because of its many influencing factors, complex symptoms, rapid changes in disease, and poor prognosis, cardiovascular disease has become the most important cause of death worldwide and has gradually begun to show increasing trends among younger people in recent years. Chemokines are a class of small molecular cytokines that can induce directed chemotaxis in response to activating G protein-coupled receptors (GPCRs). According to the arrangement of amino acid (N-terminal) cysteines, chemokines can be divided into four subgroups: CXC, CC, C and CX3C. Chemokines can be expressed by activated endothelial cells (ECs), smooth muscle cells (SMCs) and migrating leukocytes (Liu et al., 2019; Zernecke et al., 2008; Hartmann et al., 2015). To date, 17 CXC chemokines have been found in humans, most of which are involved in cardiovascular disease. CXCL1, CXCL2, CXCL3, CXCL5, CXCL6, CXCL7, and CXCL8 have proinflammatory effects, mainly through the recruitment of monocytes by CXCR2. In addition, CXCL9, CXCL10 and CXCL11 induce immune cell infiltration through CXCR3; CXCL12 recruits progenitor cells and leukocytes mainly through CXCR4, playing both proinflammatory and repair roles; and CXCL16 induces T cell recruitment by CXCR6. In addition, CXCL4 forms a heterodimer with CCL5 and induces the entry of monocytes into the endothelium. Studies of CXC chemokines associated with cardiovascular disease suggest that they play an important role in the progression of cardiovascular disease. They may therefore be potential intervention targets for multiple cardiovascular diseases.

### Characteristics of CXC Chemokines

As a class of small secreted proteins, chemokines are best known for stimulating cell migration. These chemokines, including CXCL1, CXCL2, CXCL3, CXCL5, CXCL6, CXCL7 and CXCL8, act through receptors CXCR1 and CXCR2 to mediate neutrophil function. In contrast, CXCL4 plays a role by binding to CCL5 to form heterodimers, mainly promoting monocyte recruitment (Rajarathnam et al., 2019). In addition, CXCL9, CXCL10 and CXCL11 are inflammatory chemokines that share a common receptor, CXCR3. They mainly guide the recruitment of activated T cells to exert immune functions and, to some extent, inhibit angiogenesis (Metzemaekers et al., 2017). Another study found that CXCL12, along with its two receptors CXCR4 and CXCR7, was associated with the migration of haematopoietic progenitor cells and stem cells, ECs and most leukocytes. CXCL12 mainly recruits progenitor cells and white blood cells through CXCR4, while CXCR7 mainly inhibits the CXCL12/CXCR4 axis. Additionally, CXCL12 can regulate lipid metabolism (Janssens et al., 2018; Gao et al., 2019). CXCL16 can mediate the migration of T cells through CXCR6 and can also be used as a scavenger receptor for the oxidation of low-density lipoprotein (Sheikine and Sirsjo, 2008). Chemokines play a variety of roles in inflammation, immunity, injury repair and other processes. The recruited cells and major sources of CXC chemokine family members in disease are listed in Table 1.

**TABLE 1:** Recruitment cells of CXC chemokines and their main source.

CXC chemokines	Major recruitment	Other recruitment	Main sources	Reference
CXCL1	neutrophile granulocytes	Monocytes	endothelial cells (main), clasmatoblast, macrophage, neutrophile granulocytes	Miyake et al. (2013), Girbl et al. (2018)
CXCL2	neutrophile granulocytes	—	neutrophile granulocytes (main), clasmatoblast, macrophage	De Filippo et al. (2013), Girbl et al. (2018), Lentini et al. (2020)
CXCL3	neutrophile granulocytes	Monocytes		Zhang et al. (2016)
CXCL4	Monocytes	T lymphocytes, neutrophile granulocytes	Activated platelets	Xiao et al. (2008), Schwartzkopff et al. (2012), Van Raemdonck et al. (2015), Silva-Cardoso et al. (2017)
CXCL5	neutrophile granulocytes	Monocytes	platelets, ECs	Mei et al. (2010), Rousselle et al. (2013)
CXCL6	neutrophile granulocytes	—	ECs	Gijsbers et al. (2005)
CXCL7	neutrophile granulocytes	—	monocytes, platelets	Schenk et al. (2002), Pillai et al. (2006), Wang et al., (2010)
CXCL8	neutrophile granulocytes	—	monocytes, macrophage	Lee et al. (2015), Cho et al. (2017)
CXCL9, CXCL10, CXCL11	CD4^+^ and CD8^+^ T cells	CD4^+^ CD25^+^ Foxp3^+^ regulatory T cells, natural killer T cells, NK cells	ECs(main), macrophage, neutrophile granulocytes	Miura et al. (2001), Uppaluri et al. (2008)
CXCL12	Hematopoietic stem cells and progenitor cells	endothelial cells (ECs), leukocytes	platelets, ECs	Karin, (2010), Chatterjee and Gawaz, (2013), Janssens et al. (2018b)
CXCL13	B lymphocytes	—	stromal tissue and follicular dendritic cells	Hui et al. (2015), Liu et al. (2016)
CXCL14	B-cells	THP-1 cells, activated human natural killer cells (NKs), iDCs and monocytes	epithelium, iDCs, DCs,B-Cells,activated isolated human monocytes, platelet	Meuter and Moser, (2008), Witte et al. (2017)
CXCL15	—	—	—	—
CXCL16	T cells	platelets, peripheral blood mononuclear cells (PBMCs)	vascular wall cells, leukocytes, DCs and platelets	Tabata et al. (2005), Linke et al. (2019)
CXCL17	—	—	—	—

### Signalling Pathway of CXC Chemokines

Chemokines are first expressed by activated endothelial cells (ECs), white blood cells and smooth muscle cells (SMCs), which can exist reversibly in the form of monomers and dimers (Graham et al., 2019). They are then captured by glycoaminoglycans (GAGs) on the surface of endothelial cells and presented to white blood cells as a soluble “cloud”. Among them, the affinity between the dimer and GAGs is higher (Rajarathnam et al., 2019). Eventually, chemokines bind to receptors on the corresponding cells to initiate downstream signals.

G-protein-coupled receptors (GPCRs) are the largest and most diverse group of membrane receptors in eukaryotes. Intracellular G protein is first activated upon chemokine binding to GPCR, which is a heterotrimer with alpha (α), beta (β), and gamma (γ) subunits. The combination of chemokines and GPCRs changes the conformation of GPCRs to activate the G protein. G proteins then bind GTP to activate and dissociate into α- and βγ-subunits, and the α subunit binds to adenosine cyclase and activates it under Mg^2+^ to convert ATP into cAMP. Subsequently, cAMP-dependent protein kinase A (PKA) is activated and enters the nucleus, regulating the expression of associated genes. However, chemokine-mediated chemotaxis is mainly induced through release of the βγ-subunit (Neptune and Bourne, 1997). Gβγ activates the phosphoinositide 3-kinase (PI3K) and phosphoinositol-specific phospholipase Cβ (PLC)/inositol triphosphate (IP3)/diacyl glycerol pathways (Thelen et al., 1995). The receptors, signalling pathways, and main roles of the CXC chemokine family members are shown in Table 2.

**TABLE 2:** Receptors, signaling pathways of CXC chemokines, and their role in cardiovascular disease.

CXC chemokines	Receptor	Pathways	Main role	Reference
CXCL1	CXCR1, CXCR2	PI3K/AKT	Proinflammatory effects, Promoting angiogenesis	Sotsios and Ward, (2000), Curnock and Ward, (2003), Miyake et al. (2013), Caolo et al. (2016)
		ERK1/2
CXCL2	CXCR2	PI3K/AKT	Proinflammatory effects, Promoting angiogenesis	Lentini et al. (2020)
CXCL3	CXCR2	PI3K/AKT	Proinflammatory effects	Zhang et al. (2016)
CXCL4	CXCR3	ERK1/2 MAPK	Induction of macrophage differentiation, Anti-angiogenesis	Vandercappellen et al. (2011), Van Raemdonck et al. (2015), Silva-Cardoso et al. (2017)
CXCL5	CXCR2	PI3K/AKT	Proinflammatory effects, Promoting	Mei et al. (2010)
PI3K/AKT	Angiogenesis
CXCL6	CXCR1,CXCR2	PI3K/AKT	Proinflammatory effects	Gijsbers et al. (2005)
CXCL7	CXCR2	PI3K/AKT	Proinflammatory effects	Wang et al. (2010)
CXCL8	CXCR1,CXCR2	PI3K/AKT MAPK ROS ERK	Proinflammatory effects, Promoting angiogenesis	Petreaca et al. (2007), Kim et al. (2009), Lee et al. (2015)
CXCL9	CXCR3		Immunization	Karin, (2020)
CXCL10		STAT3, STAT6	Anti-angiogenesis	
CXCL11				
CXCL12	CXCR4,CXCR7	PI3K/AKT, mTOR, NF-κB, JAK/STAT, ERK1/2	Hematopoiesis, Promoting angiogenesis, Anti-inflammatory action	Liekens et al. (2010), Janssens et al. (2018b), Mousavi, (2020)
CXCL13	CXCR5	PI3K/AKT	Anti-inflammatory action, Anti-apoptosis	Halvorsen et al. (2014)
CXCL14	CXCR4	PI3K/AKT	Immunization, Anti-angiogenesis	Lu et al. (2016)
CXCL15	—	—	—	—
CXCL16	CXCR6	PI3K/AKT	Immunization, Promoting angiogenesis	Isozaki et al. (2013), Izquierdo et al. (2014)
CXCL17	CXCR8	—	—	—

## CXC Chemokines and Cardiovascular Diseases

### CXC Chemokines and Atherosclerosis

**FIGURE 1:**
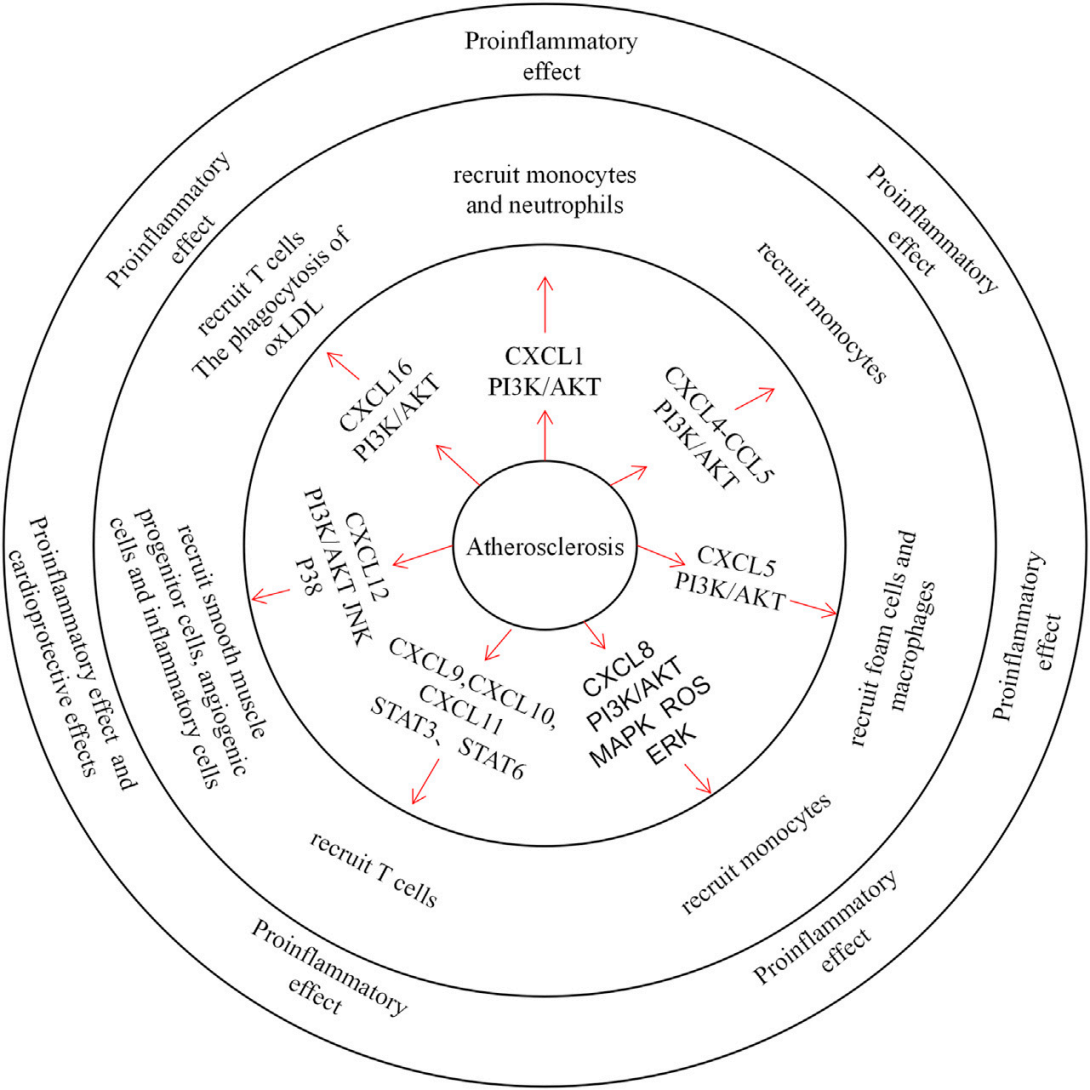
The role of CXC Chemokines in atherosclerosis.Chemokines mainly control the migration of neutrophils, monocytes, T cells, smooth muscle progenitor cells and angiogenic cells in atherosclerosis. CXCL1 and CXCL8 recruit neutrophils through the PI3K / AKT pathway and plays a pro-inflammatory role. CXCL4 forms heterodimers with CCL5 to recruit monocytes. CXCL9, CXCL10 and CXCL11 recruit T cells through STAT3, STAT6 pathway, exacerbating tissue inflammation. And CXCL12 recruits smooth muscle progenitor cells, angiogenic cells and inflammatory cells, which also plays a pro-inflammatory role and a protective role. In addition, CXCL16 recruits T cells and acts as an oxLDL clearance receptor.

Atherosclerosis is a slow progressive disease, and its initial lesions are mainly due to vascular endothelial damage and local accumulation of oxidised low-density lipoprotein (oxLDL) in the aorta. Subsequently, lipids deposited within the blood vessels and cytokines released by impaired ECs induce monocyte-directed chemotaxis (Barlic and Murphy, 2007). Foam cells are formed after oxLDL is phagocytosed by infiltrating monocytes, which is also a marker of the early course of atherosclerosis (Apostolakis et al., 2010). As a class of cytokines, CXC chemokines are involved in the process of atherosclerosis (Figure 1).

At present, research on CXC chemokines and atherosclerosis has mostly been conducted in animals, and clinical research is limited. Animal studies have shown that CXCL1 and its receptor CXCR2 are highly expressed in mouse atherosclerotic plaques. Under hypercholesterolemia, activated endothelial cells express CXCL1, which can recruit monocytes and neutrophils to the lesion through its receptor CXCR2 (Greaves et al., 2001; Soehnlein et al., 2013). Boisvert et al. established a chimeric mouse model of CXCR2 and LDLR deficiency and found that the atherosclerotic lesions of mice were reduced. CXCL1 promotes the development of atherosclerosis by regulating the migration, diffusion, and differentiation of macrophages (Boisvert et al., 2006). However, another study showed that CXCL1 stabilises plaques in the late stages of atherosclerosis. Herlea-Pana et al. found that endothelial progenitor cells (EPC) express CXCR2, the receptor of CXCL1, and can be recruited to the plaque site in the late stage of atherosclerosis to accelerate plaque regression. Under the condition of decreased blood lipids, the expression of CXCR2 on the surface of white blood cells decreased, and CXCL1 expression increased, which undoubtedly increased the recruitment effect of CXCL1 on EPCs (Yao et al., 2012; Herlea-Pana et al., 2015). These studies have shown that CXCL1 promotes inflammation early in atherosclerosis but plays a protective role in late atherosclerosis by promoting plaque stability and regression. Stable plaques generally cause stenosis or obstruction of the arteries and are rarely fatal in the absence of myocardial scarring. However, unstable plaques are prone to rupture and bleeding and cause acute cardiovascular events. It is therefore necessary to stabilise early atherosclerotic plaques (Bentzon et al., 2014).

Previous studies have shown that CXCL4 in plasma can promote the binding of monocytes to ECs by forming a heterodimer with CCL5 to enter the subendothelial space and promote atherosclerotic lesions (Domschke and Gleissner, 2019; von Hundelshausen et al., 2005). In a clinical study, researchers did not find evidence that CXCL4 levels are directly associated with coronary artery disease (CAD) (Erbel et al., 2015). However, inhibition of CCL5 with CCR5 reduced the recruitment and activation of inflammatory cells and prevented CCR5-mediated mechanical dysfunction of cardiomyocytes in the SIV/macaque model of HIV (Kelly et al., 2014).

Clinical evidence has suggested a negative correlation between CXCL5 plasma levels and CAD severity. Moreover, several CXCL5 and CXCR2 aggregates were observed in coronary atherosclerotic plaques, suggesting that CXCL5 plays a protective role in CAD (Ravi et al., 2017). Animal studies have demonstrated that foam cells and macrophages accumulate in atherosclerotic plaques and decrease collagen content in Apoe^−/−^ mice with inhibition of CXCL5. This result suggests that CXCL5 may delay the progression of atherosclerosis by limiting macrophage accumulation and foam cell formation (Rousselle et al., 2013).

Almost all nucleated cells can produce CXCL8. However, it is mainly overexpressed in diseased macrophages, ECs and SMCs (Apostolakis et al., 2009). Animal experiments have demonstrated that CXCL8 can rapidly cause rolling monocytes to adhere firmly onto monolayers expressing E-selectin, whereas related chemokines do not (Boisvert et al., 2006). Studies have shown a significant reduction in atherosclerosis in CXCL8 and LDLR deficiency, although this decrease occurs in only half of the mice with CXCR2 and LDLR deficiency. This also suggests that CXCL8 can, to some extent, promote the development of atherosclerosis through the recruitment of macrophages (Gerszten et al., 1999).

IFN-γ can stimulate endothelial cells to produce CXCL9, CXCL10, and CXCL11 to recruit and retain activated T cells at the atherosclerotic site (Mach et al., 1999). In patients with stable angina, all three chemokine levels were elevated (de Oliveira et al., 2009). Heller et al. demonstrated that CXCL10 can stimulate atherosclerosis by inhibiting the aggregation of regulatory T cells (Tregs) to lesion sites and recruiting activated T cells. CXCL9 and CXCL11 share a common receptor with CXCL10, i.e., CXCR3; thus, we speculate that they may play the same role. On the one hand, the formation of atherosclerotic lesions in mice was significantly inhibited, and cell proliferation and cell activation at the lesion site were also reduced after knockout of the CXCR3 gene (Veillard et al., 2005; Heller et al., 2006). On the other hand, using CXLCL10-neutralising antibodies to treat Apoe^−/−^ mice with unstable plaques, Dolf Segers et al. found that the atherosclerotic plaques were more stable. Additionally, in human arterial intima specimens, they observed that higher human plasma CXCL10 levels correlated with more unstable plaques. All of this evidence suggests that CXCL10 may be positively associated with unstable atherosclerotic plaques (Segers et al., 2011).

The role of CXCL12 in atherosclerosis is controversial, although there is substantial evidence indicating that CXCL12 plays a protective role. Through the use of intravenous CXCL12 to treat Apoe^−/−^ mice, Akhtar et al. observed that the fibrous cap of diseased plaques in mice was thickened and that smooth muscle cells increased, but the lesion size did not change significantly (Akhtar et al., 2013). The injured endothelial cells release apoptotic bodies to induce peripheral vascular cells to produce CXCL12, which can recruit smooth muscle progenitor cells and promote atherosclerotic stable plaque formation (Zernecke et al., 2009). Plasma CXCL12 levels in CAD patients were lower than those in healthy individuals, and plasma CXCL12 levels in advanced atherosclerotic mice were also lower than those in normal mice. This suggests that CXCL12 may have antiatherosclerotic effects (Damas et al., 2002; Xu et al., 2011). Mice treated with the CXCR4 inhibitor AMD3465 had increased lesions and leucocytosis in the plaque, suggesting that CXCL12 might resist atherosclerosis by regulating neutrophil release. Zernecke et al. also found that CXCL12 can protect endothelial integrity via CXCR4 through recruitment of angiogenic cells (Zernecke et al., 2008). Additionally, there is evidence that CXCL12 has proatherogenic effects. According to epidemiological investigations, CXCL12 levels were positively correlated with the risk of CAD onset (Sjaarda et al., 2018). Reduced aortic lesions were observed in mice with arterial endothelial (EC)-specific CXCL12 deficiency, suggesting that CXCL12 from ECs can promote atherosclerosis (Doring et al., 2019). Ma et al. found that CXCL12 can promote macrophage phagocytosis by activating its other receptor, CXCR7, which activates the JNK and P38 pathways and leads to atherosclerosis (Ma et al., 2013). CXCL12 can also promote neointimal formation by recruiting smooth muscle progenitor cells and stimulating vascular smooth muscle cell (VSMC) proliferation. After treating mice with the CXCL12 antagonist NOX-A12, both intralesion SMCs and neointimal hyperplasia were observed (Thomas et al., 2015; Zernecke et al., 2005). Moreover, CXCL12 can also recruit EPCs to promote neovascularization in injured arteries (Kanzler et al., 2013). An increase in CXCL12 expression, which can cause platelet aggregation and prolong survival of the thrombus, was found in patients with angina pectoris (Kraemer et al., 2010; Ohtsuka et al., 2017). Neointima formation, neoangiogenesis and platelet aggregation all aggravate atherosclerosis.

Clinical studies have shown a significant increase in serum CXCL16 concentrations in patients with atherosclerosis (Wang et al., 2010). The CXCL16 gene polymorphism rs3744700 is closely related to coronary heart disease and can increase the risk of coronary heart disease (Tian et al., 2015). Another study showed that CXCL16 levels were significantly positively correlated with the severity of coronary atherosclerotic heart disease (Xing et al., 2018). Many studies have found that, when CXCL16 levels increase, the probability of poor prognosis in patients with coronary syndrome also increases (Jansson et al., 2009; Andersen et al., 2019). Soluble SR-PSOX/CXCL16 is significantly reduced in acute coronary syndrome, and its specificity and sensitivity are higher than those of high-sensitivity C-reactive proteins. Therefore, Mitsuoka et al. proposed that soluble SR-PSOX/CXCL16 could serve as a biomarker for ACS (Mitsuoka et al., 2009). CXCL16 can recruit T cells expressing CXCR6 and promote local inflammation aggravation. Moreover, mice lacking CXCR6 showed reduced lesions (Zernecke et al., 2008). In addition, CXCL16 acts as an oxLDL clearance receptor to fight atherosclerosis, and the phagocytosis of oxLDL by macrophages in CXCL16-deficient mice was decreased. Furthermore, CXCL16^−/−^/LDLR^−/−^ mice showed accelerated atherosclerotic lesions. This evidence confirms the antiatherosclerotic effects of CXCL16 (Aslanian and Charo, 2006).

### CXC Chemokines and Myocardial Infarction and Cardiac Ischaemia Reperfusion Injury

**FIGURE 2:**
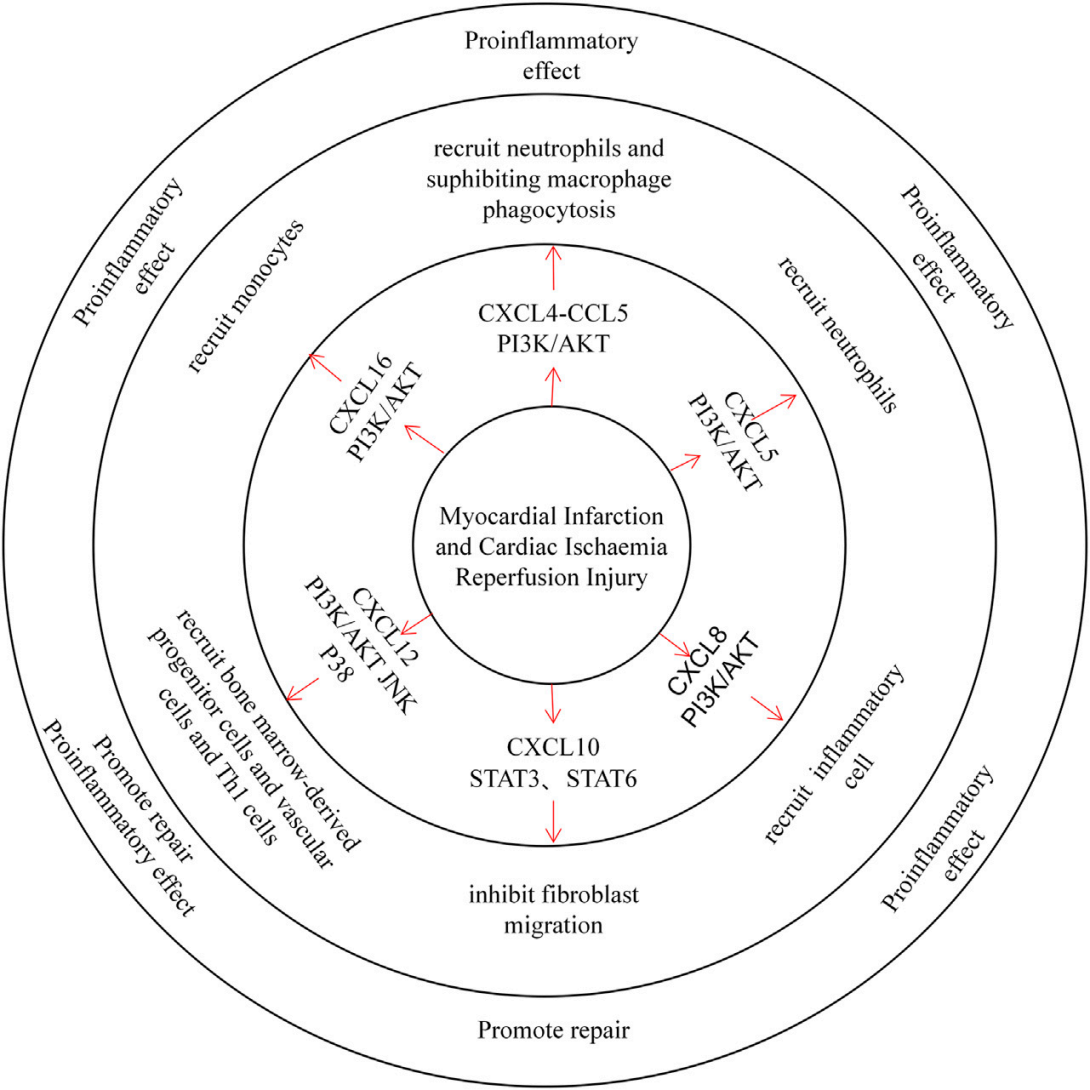
CXC chemokines in the Myocardial Infarction and Cardiac Ischaemia Reperfusion Injury. CXCL4 recruited neutrophils in a mouse MI model and inhibited macrophage phagocytosis after MI, exacerbating tissue injury. CXCL5 recruits neutrophils and promotes inflammatory development. CXCL8 recruits inflammatory cells through the PI3K / AKT pathway and plays a pro-inflammatory role. CXCL10 can inhibit fibroblast migration and thus promote tissue repair. CXCL12 recruits both bone marrow-derived progenitors, vascular cells, and Th1 cells, playing both proinflammatory and repair roles. CXCL16 primarily recruits monocytes to promote inflammatory development.

Myocardial infarction (MI) occurs mostly in patients with coronary heart disease and is caused by myocardial ischaemia and hypoxia, which are caused by coronary artery occlusion. Injured cardiomyocytes can activate complement, produce reactive oxygen species and induce cytokine upregulation. Upregulated cytokines then recruit leucocytes to the injured site, exacerbating inflammation (Figure 2) (Frangogiannis, 2014; Lu et al., 2015).

CXCL4 itself has a strong proinflammatory effect. Nevertheless, in the I/R model, Vajen et al. observed a decrease in the area of MI and a decrease in the number of neutrophils in the infarct area by blocking the isomerization of CXCL4 with CCL5. Lindsey et al. also found that infusion of exogenous CXCL4 into MI mice inhibited phagocytosis of macrophages and increased mortality after MI (Vajen et al., 2018; Koenen, 2019; Lindsey et al., 2019). CXCL5 expressed by cardiomyocytes is upregulated in a mouse ischaemia-reperfusion model, while CXCL5 can recruit neutrophils to aggravate myocardial ischaemia-reperfusion injury (Chandrasekar et al., 2001). Furthermore, studies have demonstrated that CXCL8 is upregulated by Ang II in the infarcted myocardium, similarly inducing inflammatory cell infiltration (Nabah et al., 2004; Sun et al., 2019). The use of FR183998 in reperfusion models significantly inhibited the content of CXCL8 and the occurrence of MI (Ohara et al., 2002). According to these studies, CXCL8 can promote MI in the case of myocardial ischaemia. CXCL10 is similarly upregulated in the infarcted myocardium, and animal experiments have shown that CXCL10-deficient mice over-repair and have scarred cardiomyocytes after reperfusion. Furthermore, CXCL10 can inhibit fibroblast migration while promoting wound contraction, playing a protective role in MI (Bujak et al., 2009). The role of CXCL12 in MI is controversial. On the one hand, a great deal of evidence suggests an increase in plasma CXCL12 in patients with myocardial infarction (Zhuang et al., 2009; Kim et al., 2016). In the case of heart damage, CXCL12 can protect cardiomyocytes from IRI damage and improve the proliferation of cardiomyocytes (Bromage et al., 2014; Hou et al., 2015). In the early stages of MI, CXCL12 can recruit bone marrow-derived progenitor cells and vascular cells in the heart, promoting cardiovascular production and cardiac repair (Ghadge et al., 2011; Goldstone et al., 2018). Experiments have shown that the left ventricular MI of mice treated with SDF-1αPEG fibrin patches is better than that of the control group. This suggests that increased local release of CXCL12 can increase stem cell homing and repair damaged hearts (Zhang et al., 2007). On the other hand, experiments have shown that CXCL12 has adverse effects on MI. Mice overexpressing CXCL12 had impaired cardiac function and increased myocardial fibrosis after MI, and high concentrations of CXCL12 can upregulate TNF-α protein to induce cardiomyocyte apoptosis. In addition, CXCL12-deficient mice showed retention of cardiac function and decreased Th1 cell infiltration. This indicates that CXCL12 can aggravate MI by promoting Th1 cell infiltration, cardiomyocyte apoptosis and cardiac fibrosis (Muhlstedt et al., 2016; Jarrah et al., 2018). In the ischaemia-reperfusion model, therapeutic CXCL12 2 hours in advance reduced the myocardial infarction area in mice. This evidence shows that CXCL12 can have a protective effect (Bromage et al., 2019). However, another experiment found that treatment of mice after ischaemia/reperfusion injury with the CXCR4 inhibitor AMD3100 significantly improved cardiac function after reperfusion. This could be due to the inhibition of CXCL12-mediated recruitment of CXCR4^+^ inflammatory cells (Chen et al., 2010; Jujo et al., 2013; Wang et al., 2019). These opposing results may have been due to the different models used by the two experiments. CXCL12 transgenic overexpressing (Tg) rats promoted inflammation and fibrosis after the induction of myocardial infarction. Another set of experiments using fibrin patches to control the release of CXCL12 to the MI site in mice increased the recruitment of c-kit^+^ cells to the mouse heart and significantly improved cardiac function. This indicates that the early administration of exogenous CXCL12 may improve cardiac function after myocardial infarction. Plasma CXCL16 levels were similarly elevated in myocardial infarction mice, as CXCL16 exerts a protective function by promoting macrophage phagocyte fragments (Xiao et al., 2014). CXCR6 KO mice showed a smaller infarct size and better cardiac function under I/R induction. This finding indicates that failure of the CXCL16-CXCR6 axis can resist I/R damage (Zhao et al., 2013).

### CXC Chemokines and Hypertension

Hypertension is a serious public health problem worldwide. During hypertension, infiltration of immune cells often leads to tissue damage and elevated blood pressure. The injured tissue further releases IFN-γ to promote T lymphocyte migration. Moreover, injured tissues express several kinds of CXC chemokines (such as CXCL1-CXCL8); regulate the accumulation of neutrophils; and promote vascular inflammation, dysfunction and injury (Rudemiller and Crowley, 2017).

A recent study showed elevated blood CXCL1 and CXCL2 levels in spontaneously hypertensive rats (SHRs). Treatment with CXCR2 inhibitors inhibits the accumulation of monocytes/macrophages and reduces the production of proinflammatory cytokines and ROS, thereby weakening cardiac remodelling and improving cardiac function (Zhang et al., 2019; Zhang et al., 2020). Upon hypertension, AngII mediates continuous expression of CXCL8 through the AT1 receptor. Meanwhile, CXCL8 promotes VSMC proliferation through the ERK pathway and increases hypertension (Kim et al., 2008; Kim et al., 2009). Stimulated by hypertension, the tissue may secrete various inflammatory factors, including CXCL10. This view is supported by increased circulating levels in patients with hypertension. CXCL10 induces infiltration of T cells in the kidney, causing T cell-driven inflammation and exacerbating hypertension and kidney damage (Antonelli et al., 2012; Youn et al., 2013). Similarly, AngII induced CXCL16 expression in renal tubular epithelial cells by activating NF-κB. Experiments have shown that the absence of CXCL16 inhibits the recruitment of bone marrow-derived fibroblasts, macrophages, and T cells into the kidney and reduces fibrosis of the renal interstitium (Xia et al., 2013; Ma et al., 2016).

### CXC Chemokines and Aortic Aneurysms and Aortic Dissection

Both aortic aneurysm and aortic dissection are associated with the degeneration of aortic elastic mediators, especially loss of SMCs. Among them, the production of reactive oxygen species and inflammatory factors is closely related to the apoptosis of SMCs. Loss of SMCs destroys the integrity of the aortic structure and further exacerbates both lesions (Lopez-Candales et al., 1997; Sakalihasan et al., 2005).

Clinical studies have shown that serum CXCL8 levels in patients with abdominal aortic aneurysms (AAAs) are increased, and neutrophils are recruited. Neutrophil release of multiple matrix degradation proteases (MMPs) induces destruction of aortic extracellular matrix components and VSMC apoptosis in human AAA (Kokje et al., 2018). Another study showed elevated CXCR3 levels in thoracic aortic aneurysms. Animal experiments have shown that CXCR3 is closely related to mouse aneurysms and can promote aneurysm formation (Gallo et al., 2012). Only one study mentioned that upregulation of CXCL10 expression via IFN-γ induction can inhibit the formation and rupture of AAAs. CXCL10 can raise T lymphocyte levels and reduce the enrichment of non-Th1 cytokines at the lesion site, including transforming growth factor-β1 (TGF-β1). Thus, CXCL10 reduces the expansion of aneurysms and delays disease progression by inhibiting TGFβ1-mediated VSMCs (King et al., 2009).

Parietti et al. found a positive correlation between CXCL12 levels and aortic aneurysm size (Parietti et al., 2011). The expression of CXCL12 and CXCR4 genes was significantly increased in AAA, especially CXCR4 in neutrophils (Tanios et al., 2015). Blocking CXCR4 with AMD3100 reduced the infiltration of outer membrane macrophages in experimental AAA and significantly inhibited AAA amplification. This finding suggests that CXCL12 likely aggravates AAA lesions through proinflammatory effects (Michineau et al., 2014). Another study showed that the CXCL12/CXCR4 axis could induce homing of rat bone marrow mesenchymal stem cells (BMSCs) and delay further AAA development (Long et al., 2014). With regard to aortic dissection, only one study showed that CXCL1 levels increased after abdominal aortic dissection (AAD), promoting neutrophil infiltration. Neutrophils can express high levels of IL-6, leading to outer membrane inflammation with dilation and rupture of the aortic arch (Anzai et al., 2015).

### CXC Chemokines and Cardiac Fibrosis

**FIGURE 3:**
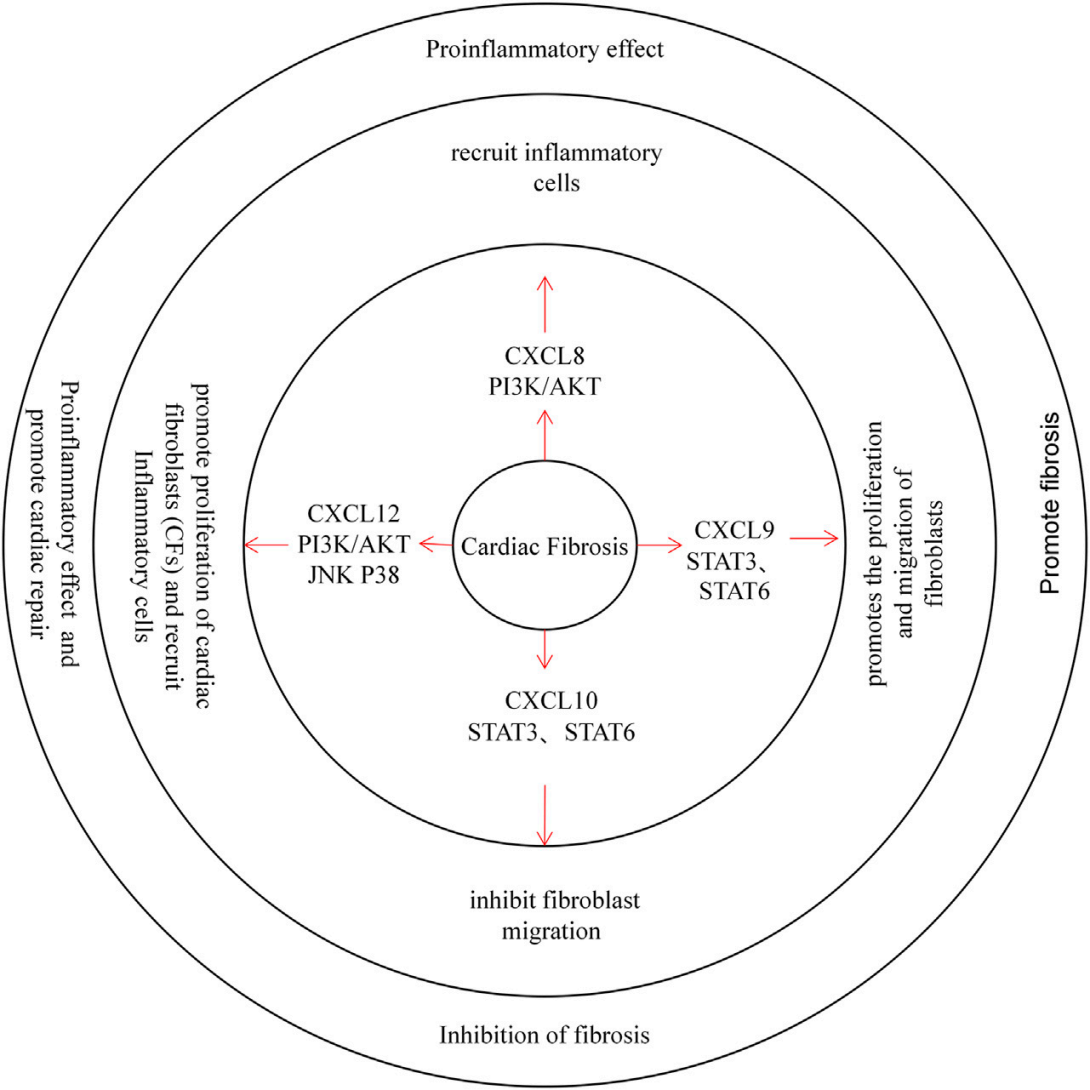
CXC chemokines in Cardiac Fibrosis. CXCL8 recruits inflammatory cells through the PI3K / AKT pathway and plays a pro-inflammatory role. CXCL9 promotes the proliferation and migration of fibroblasts through the STAT3 and STAT6 pathway, promoting cardiac fibrosis. Instead, the CXCL10 inhibit fibroblast migration and protects the heart from Cardiac Fibrosis. CXCL12 can promote the proliferation of cardiac fibroblasts (CFs), and recruit inflammatory cells, while exerting pro-inflammatory and repair effects.

Myocardial fibrosis is caused by excessive repair of damaged myocardium. Impaired apoptosis of cardiomyocytes induces the production of a large number of cytokines and promotes the proliferation of myocardial fibroblasts. At the same time, fibroblasts synthesize a large number of collagen fibres to accelerate the repair of damaged tissues, causing heart fibrosis (Figure 3) (Dobaczewski and Frangogiannis, 2009).

Few studies have evaluated CXC chemokines and cardiac fibrosis. One study mentioned that CXCR4 antagonists could delay cardiac fibrosis in mice with type I and II diabetes. In addition, treatment with CXCL12 can cause proliferation and hypertrophy of cardiac fibroblasts (CFs) in mice and promote CFs to produce collagen. This result was more significant in hypertension models (Jackson et al., 2017; Wang et al., 2020). Moreover, CXCL12 itself can promote cardiac repair and delay cardiac fibrosis. After inhibiting the scavenger receptor CXCR7, the fibrosis process slowed in mice (Chu et al., 2015). The effect of CXCL12 on promoting cardiac fibrosis may be due to the recruitment of CXCR4-expressing inflammatory cells that trigger local inflammation and CF activation (Menhaji-Klotz et al., 2018). CXCL8 plays a proinflammatory role in cardiac fibrosis, and increased CXCL9 expression after MI promotes the proliferation and migration of fibroblasts. Unlike CXCL9, however, CXCL10 can inhibit fibroblast migration (Dobaczewski and Frangogiannis, 2009; Turner et al., 2011; Lin et al., 2019).

### CXC Chemokines and Atrial Fibrillation

**FIGURE 4:**
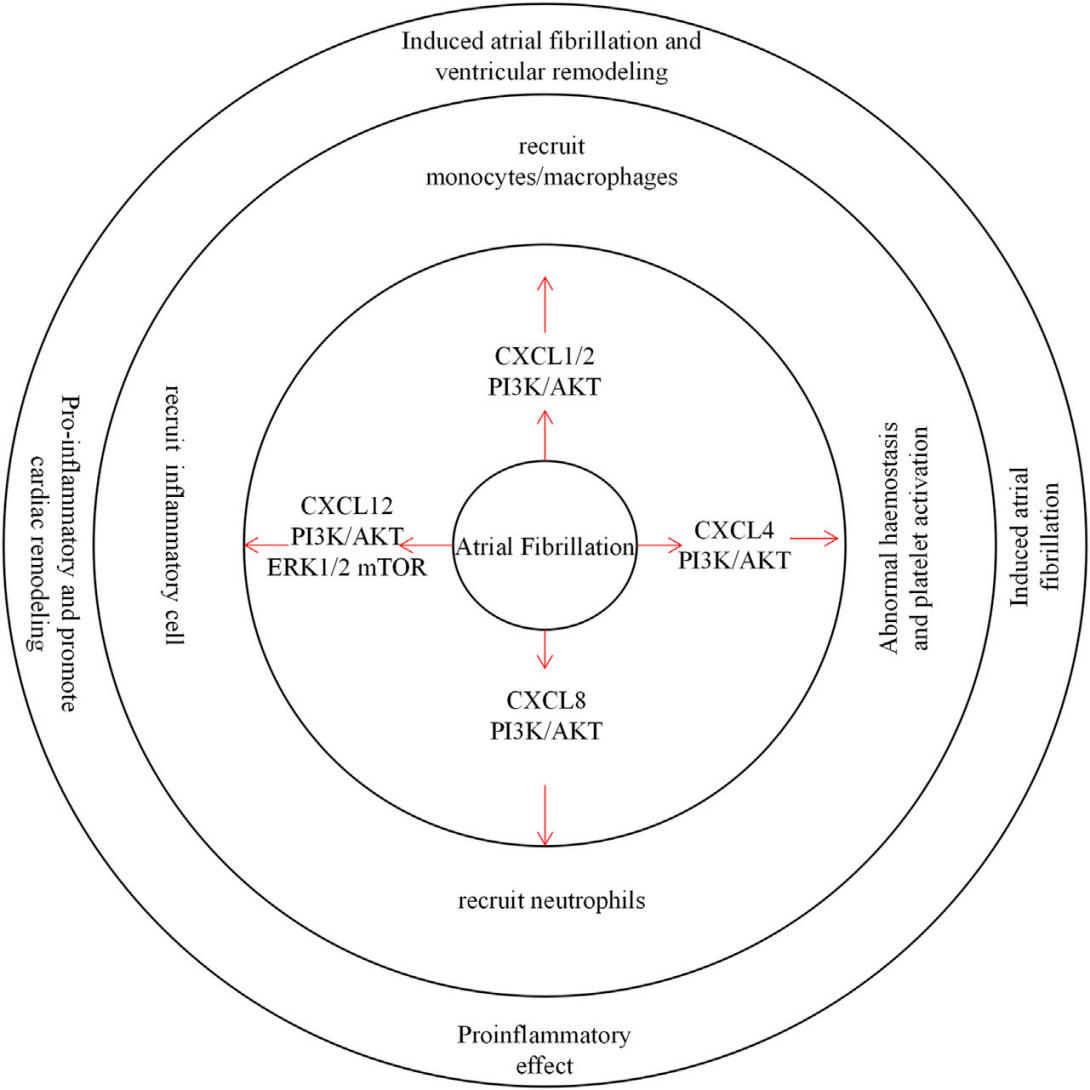
CXC chemokines in Atrial Fibrillation. CXCL1/2 recruits monocytes/macrophages through the PI3K / AKT pathway to induce atrial fibrillation and ventricular remodeling. CXCL4 is involved in hemostasis and abnormal platelet activation, inducing the development of atrial fibrillation. CXCL8 recruits inflammatory cells through the PI3K / AKT pathway and plays a pro-inflammatory role. CXCL12 recruits inflammatory cells and promotes inflammation associated with cardiac remodeling.

Atrial fibrillation (AF) is a common arrhythmia that is often closely associated with inflammation and atrial fibrosis (Figure 4) (Melenovsky and Lip, 2008).

There is growing evidence that inflammatory cells, especially monocytes/macrophages, play an important role in AF. CXCL1/2 regulate the entry of CXCR2^+^ monocytes/macrophages into cardiac tissues and lead to the further development of AF. In contrast, inhibition of the CXCR2-MAPK (mitogen-activated protein kinase) and nicotinamide adenine dinucleotide phosphoroxidase, NF-κB, and TGFβ-1/Smad2/3 pathways significantly attenuated atrial infiltration in monocytes/macrophages, AF induction, and atrial remodelling in Ang II-infused mice (Zhang et al., 2020). The inflammation resolution-promoting molecule resolvin-D1 reduced CXCL1 and CXCL2 expression in heart tissues of monocrotaline MCT-treated rats and simultaneously attenuated AF induction in MCT rats and reduced the mean AF duration (Hiram et al., 2021). Abnormal haemostasis and platelet activation occurs in permanent AF patients, and Serkan et al. observed high levels of CXCL4 in the plasma of AF patients (Kamath et al., 2002; Topaloglu et al., 2007). A clinical report showed that elevated blood CXCL8 levels in patients with coronary artery bypass transplantation (CABG) were associated with the occurrence of atrial fibrillation. CXCL8 may be produced after reperfusion of ischaemic myocardium (Melenovsky and Lip, 2008; Wu et al., 2008). CXCL12 expression was also increased, especially in patients with permanent and persistent AF. Another clinical study showed a similar increase in CXCR4 expression in AF patients (Stellos et al., 2012). In an AF model, CXCR4 inhibitors blocked hyperactivation of ERK1/2/AKT/mTOR signalling in the atrium, reduced atrial inflammation, and delayed left ventricular remodelling (Larocca et al., 2019; Liu et al., 2021).

### CXC Chemokines and Rejection After Heart Transplantation

**FIGURE 5:**
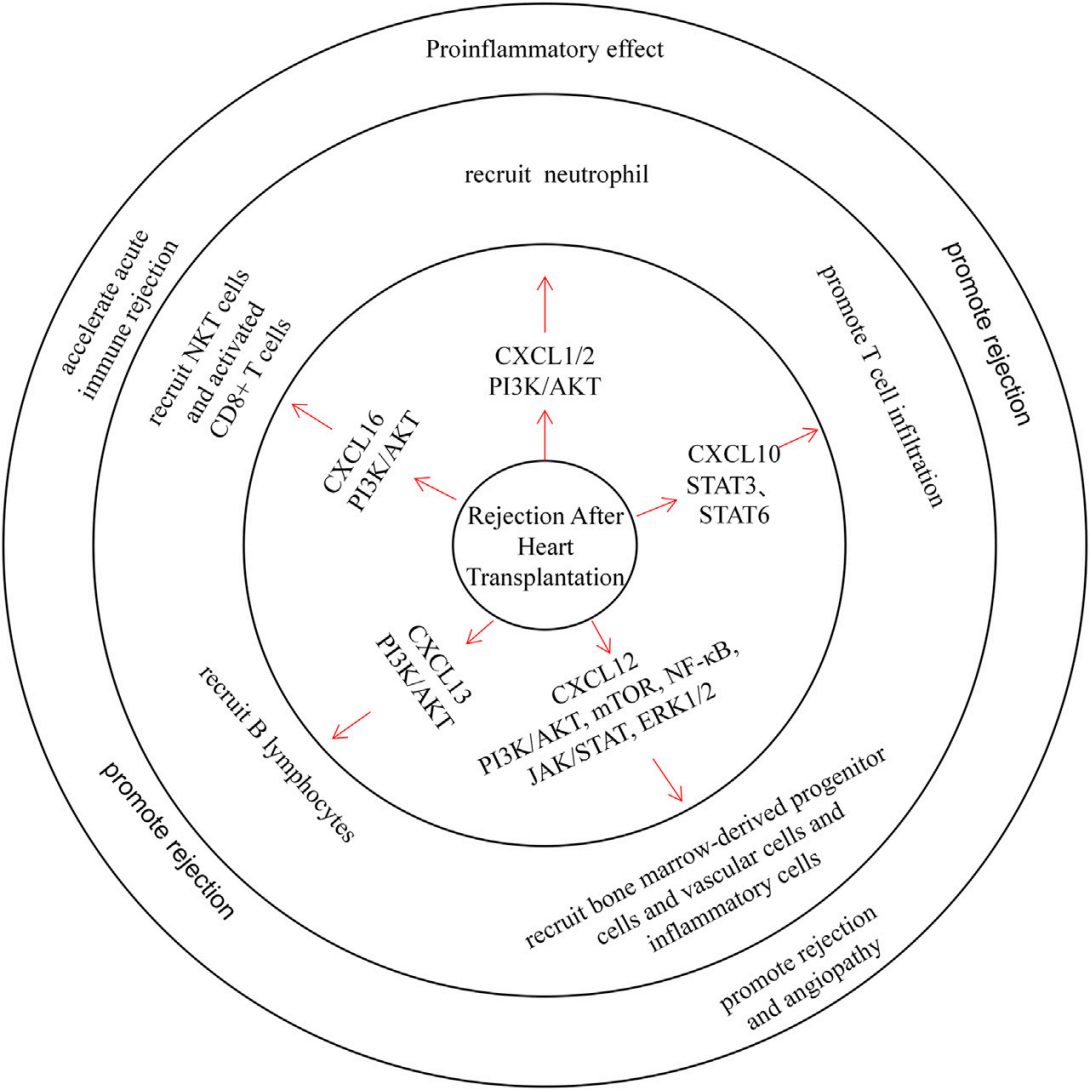
CXC chemokines in Rejection After Heart Transplantation. CXCL1/2 recruits neutrophils and plays a pro-inflammatory role. CXCL10 can promote T cell infiltration and promote rejection. CXCL12 recruits bone marrow-derived progenitor cells, vascular cells, and inflammatory cells through multiple pathways, promoting rejection and angiopathy. CXCL13 recruits B lymphocytes and promotes rejection. CXCL16 recruits NKT cells and activated CD8+ T cells, which can accelerate acute immune rejection.

Rejection is an important complication of heart transplantation, and recruitment of lymphocytes to the transplanted heart leads to organ structure damage, the basis of rejection (Figure 5) (Long et al., 2014).

Early CXCL1 and CXCL2 expression was increased in blood upon neutrophil infiltration in heart transplant mice (Fairchild et al., 1997; Yun et al., 2000), and treating heart transplant mice with CXCL1/CXCL2 antibodies can prolong heart transplant survival. Wieder et al. also found that the treatment of heart transplant rats with rapamycin prolonged graft survival, and they observed a decrease in CXCL1/CXCL2 content and neutrophil infiltration in these rats (Wieder et al., 1993; El-Sawy et al., 2005).

Previous clinical studies have reported elevated CXCL9 and CXCL10 levels after cardiac transplantation (Ma et al., 2015). After a postoperative follow-up survey of heart transplant patients, Michael et al. found that CXCL10 was significantly induced upon acute rejection, and expression of its receptor CXCR3 was also associated with T cell infiltration (Melter et al., 2001). Moreover, plasma CXCL10 levels decreased in patients who were treated with simvastatin. These data also further confirm that CXCL10 promotes rejection (Nykanen et al., 2019). Animal experiments have shown that treating heart transplant mice with CXCL9 and CXCL10 inhibitors reduces memory T lymphocyte infiltration in the graft and prolongs its survival (Yun et al., 2002; Ma et al., 2015). In a previous study, Michael et al. reported that inhibition of the CXCL12/CXCR4/CXCR7 axis could improve chronic rejection after heart transplantation in mice (Michael et al., 2015). Furthermore, a combination of CXCR4 antagonists and immunosuppressive agents can reduce rejection and angiopathy in a porcine heart transplant model (Hsu et al., 2018). All of these results demonstrate the effect of CXCL12 on rejection after heart transplantation. Moreover, CXCL13 can recruit B lymphocytes through CXCR5 and induce acute immune rejection (Di Carlo et al., 2007). Similarly, in allograft transplantation, CXCL16 can recruit NKT cells and activated CD8^+^ T cells through CXCR6 and accelerate acute immune rejection (Jiang et al., 2005; Jiang et al., 2010).

### CXC Chemokines and Other Cardiovascular Diseases

CXC chemokines are also involved in other types of heart disease, such as cardiomyopathy, viral myocarditis, congenital heart disease and ventricular fibrillation (Delete: However, there are few reports about them).

Anna et al. reported that CXCL1 knockout mice were more likely to survive bur-type spirochete-induced myocarditis, and reduced neutrophil infiltration at the lesion site reduces heart disease (Ritzman et al., 2010). In addition, in acute stress (Takotsubo) cardiomyopathy, CXCL1 expression was upregulated, and the number of monocytes was increased (Scally et al., 2019). In viral myocarditis, Coxsackie virus B type 3 (CVB3) infection induces CXCL2 and CXCL10 expression in myocardial tissue. An upregulation of CXCL10 expression and a decrease in viral titre were also observed in early stage viral myocarditis, indicating that CXCL10 plays a protective role in viral myocarditis (Shen et al., 2003; Yuan et al., 2009). Moreover, studies have shown that cardiomyocyte-specific CXCR4 knockout (CXCR4cKO) mice exhibit progressive cardiomyopathy and that CXCL12 treatment prevents isoproterenol-induced cardiac hypertrophy (Larocca et al., 2019). Another experiment demonstrated that CXCL12 can promote cardiac fibrosis in dilated cardiomyopathy mice (Chu et al., 2019). According to clinical reports, CXCL16 expression is upregulated in patients with inflammatory cardiomyopathy and heart failure (Dahl et al., 2009; Borst et al., 2014). CXCL16 is also elevated in inflammatory valvular heart disease, which mediates the adhesion of CD8^+^ T cells to ECs through VLA-4 and stimulates CD8^+^ T cells to produce IFN-γ (Yamauchi et al., 2004). The expression of chemokines varies in cardiovascular disease in human and mouse, and these differences are summarized in Table 3.

**TABLE 3:** Expression of several common CXC chemokines in cardiovascular diseases.

	Disease	CXCL1	CXCL8	CXCL9	CXCL10	CXCL11	CXCL12	CXCL16	References
Mice	Atherosclerosis	Increase	Increase	Increase	Increase	Increase	Decrease	—	Boisvert et al. (2006), Gerszten et al. (1999), Heller et al. (2006), Xu et al. (2011)
	Hypertension	Increase	Increase	—	—	—	—	Increase	Zhang et al. (2019), Kim et al. (2008), Li et al. (2018), Ma et al. (2016)
	Aortic Aneurysms	—	Increase	Decrease	Decrease	Decrease	Increase	—	Kokje et al. (2018), Gallo et al. (2012), Michineau et al. (2014)
	Myocardial Infarction	—	Increase	Increase	Increase	Increase	Increase	Increase	Sun et al. (2019), Bujak et al. (2009), Bromage et al. (2019), Xiao et al. (2014)
	Rejection After Heart Transplantation	Increase	—	Increase	Increase	Increase	Increase	Increase	Yun et al. (2000), Ma et al. (2015), Michael et al. (2015), Jiang et al. (2005)
Human	Atherosclerosis	Increase	Decrease	Increase	Increase	Increase	Decrease	Increase	Greaves et al. (2001), Apostolakis et al. (2009), de Oliveira et al. (2009), Damas et al. (2002)
	Hypertension	—	—	Increase	Increase	Increase	Increase	Increase	Antonelli et al. (2012), Xia et al. (2013), Mccullagh et al. (2015)
	Aortic Aneurysms	—	Increase	—	—	—	Increase	—	Kokje et al. (2018), Parietti et al. (2011)
	Myocardial Infarction	—	Increase	—	—	—	Increase	—	Nabah et al. (2004), Zhang et al. (2007)
	Rejection After Heart Transplantation	—	—	Increase	Increase	Increase	—	Increase	Ma et al. (2015), Jiang et al. (2010)

## Discussion

We attempted to describe the expression, signalling pathways, sources and main roles of CXC chemokines in different cardiovascular diseases. First, CXCL1, CXCL2, CXCL3, CXCL5, CXCL6, CXCL7 and CXCL8 can recruit neutrophils through the common receptor CXCR2; play a proinflammatory role; and promote angiogenesis to some extent. Moreover, CXCL1, CXCL6, and CXCL8 can also recruit inflammatory cells through CXCR1 to promote the development of inflammation. However, CXCL4 is special in cardiovascular disease, as it needs to form a heterodimer with CCL5 to promote monocyte adhesion and play an antiangiogenic role. In addition, CXCL9, CXCL10, and CXCL11 share the same receptor, CXCR3. They promote cellular immunity by recruiting T cells and are often upregulated in heart transplantation and viral infections. Unlike previous CXC chemokines, CXCL12 mainly recruits haematopoietic stem and progenitor cells and can promote the repair of haematopoietic and damaged tissues. However, two roles have been proposed for CXCL12 in cardiovascular disease. CXCL12 can recruit smooth muscle progenitor cells and endothelial progenitor cells through CXCR4, promote the repair of damaged tissues, or recruit inflammatory cells to a certain extent to play a proinflammatory role. In contrast, CXCL14 recruits B lymphocytes with natural killer cells through CXCR4 to play an immune role. CXCL16 also enhances cellular immunity by promoting the adhesion of T cells and some peripheral blood monocytes to endothelial cells.

Here, we report a diverse role of CXC chemokines in cardiovascular disease. Blocking the CXCR2 pathway primarily inhibits the development of cardiac inflammation and may help to improve the prognosis of inflammation-related cardiovascular diseases. In contrast, promoting the antiangiogenic effects of CXCL4 can inhibit tumour development to some extent. The CXCL9,10,11/CXCR3 axis can also play an antitumour role. On the other hand, inhibition of this axis can attenuate the occurrence of immune rejection. This may be a potential target for therapeutic intervention after heart transplantation. Furthermore, inhibition of the CXCL12/CXCR4 axis can improve cardiac fibrosis and promote tissue repair after myocardial infarction. CXC chemokines therefore play an important role in cardiovascular disease and may be potential intervention targets for multiple cardiovascular diseases. However, further research is needed.
